# Rank Difference Analysis of Microarrays (RDAM), a novel approach to statistical analysis of microarray expression profiling data

**DOI:** 10.1186/1471-2105-5-148

**Published:** 2004-10-11

**Authors:** Dietmar E Martin, Philippe Demougin, Michael N Hall, Michel Bellis

**Affiliations:** 1 Biozentrum, University of Basel, CH-4056 Basel, Switzerland; 2 CRBM, FRE2593, CNRS, Montpellier, France

## Abstract

**Background:**

A key step in the analysis of microarray expression profiling data is the identification of genes that display statistically significant changes in expression signals between two biological conditions.

**Results:**

We describe a new method, Rank Difference Analysis of Microarrays (RDAM), which estimates the total number of truly varying genes and assigns a p-value to each signal variation. Information on a group of differentially expressed genes includes the sensitivity and the false discovery rate. We demonstrate the feasibility and efficiency of our approach by applying it to a large synthetic expression data set and to a biological data set obtained by comparing vegetatively-growing wild type and tor2-mutant yeast strains. In both cases we observed a significant improvement of the power of analysis when our method is compared to another popular nonparametric method.

**Conclusions:**

This study provided a valuable new statistical method to analyze microarray data. We conclude that the good quality of the results obtained by RDAM is mainly due to the quasi-perfect equalization of variation distribution, which is related to the standardization procedure used and to the measurement of variation by rank difference.

## Background

In a typical microarray experiment, thousands of genes have their relative expression levels measured in parallel under different biological states [1,2]. To identify differentially-abundant genes, most published methods [3-5] progress through a similar sequence of elementary steps. First, a normalizing procedure is applied to make data sets comparable. If certain experimental conditions comprise several replicates, methods based either on parametric or nonparametric tests usually reduce the number of values generated by using their means. Then, gene variation is quantified by a statistic derived from intensity measurements. Knowledge of the null distribution of the gene variation, which is the distribution of its statistic when only random fluctuations occur, allows p-values to be assigned to observed variations and genes to be ranked according to the significance of their variation. As the test is repeated as many times as there are genes, p-values are corrected accordingly, and the false discovery rate (FDR, "the expected proportion of false positives among all genes declared significantly differentially expressed"[6]) is estimated.

We describe here, in detail, a new analysis method that has been used to analyze the transcriptome in yeast [7]. This method is original in several respects. First, Rank Difference Analysis of Microarrays (RDAM) replaces raw signal by its rank (R), expressed on a 0–100 scale, and we show that this simple transformation is a powerful normalizing procedure. Also, RDAM does not reduce replicated signals to their means, but instead only considers variations, expressed as rank differences (RD), between individual experimental points. An essential step is the standardization of RD observed between two replicates, permitting easy access to the empirical null distribution and allowing accurate and precise p-values to be assigned to observed standardized RD (zRD). When dealing with replicated points, RDAM uses a random variable, the product of p-values (ppv), for which the null distribution is straightforward to compute in a manner that is independent of the experimental conditions. Finally, RDAM estimates the total number of truly varying genes (TV), assigns a p-value to each gene variation, characterizes the selection of a gene using the FDR and the percentage of truly varying genes included in the selection (sensitivity, S).

Analysis of synthetic data sets allowed us to specify the error distribution of all the estimators used (FDR, TV and S), and to demonstrate the strong predominance that the number of varying genes and the distribution of their variation have on the quality of the results.

We also analysed the transcriptional effects of the TOR2-controlled signaling function using a genome-wide microarray approach in yeast. In *S. cerevisiae*, TOR2 has two essential signaling functions. One, shared with TOR1, is required for translation initiation, transcription, and cell growth in response to the presence of nutrients [8-10]. The second is unique to TOR2, and functions in cell-cycle-dependent actin polarization and possibly in transcription [8,11]. A previous genetic screen for mutants defective in the TOR-shared and the TOR2-unique functions identified several TOR2 temperature-sensitive alleles [12]. In this study, we compared total transcription profiles for strain SH121, which is specifically defective in the TOR2-unique function, and its isogenic wild type counterpart SH100 [12].

## Results

### Standardization of positive variations

The simplest system to which our method can be applied comprises three experimental points, of which two are replicates, as described by the expression {Exp1A, Exp1B, Exp2A}, where the number refers to the biological condition and the final letter refers to the replicates. To identify significant variations in the comparison Exp2A vs. Exp1A, we have to first calculate the variation of gene i, VARi. From among several possibilities, we tested three different variation units: the fold change (FC), corresponding to the ratio of signals, the signal difference (SD), and the rank difference (RD). The RD uses a standardized signal measure that is independent of the scanner settings, because the signal is replaced by its rank, expressed on a 0–100 scale. This normalizing procedure consists of first calculating the absolute rank (AR) of each gene by ordering their signals from 0 to N (with the signals of all genes having a negative signal being set to zero, and N representing the number of non-null and non-negative signals) and then transforming the absolute rank value into a relative one (R = AR*100/N). In this way, all the signals are expressed on the same scale and are directly comparable.

We studied the variation distribution between the two replicates, i.e. Exp1A and Exp1B, reasoning that the observed empirical variation distribution would be an excellent approximation of the null distribution corresponding to the null hypothesis. The null hypothesis we have in mind states that all observed mRNA changes occurring under replicated conditions are due to a combination of biological and technological noise, and are not the result of any biologically significant process.

**Figure 1:**
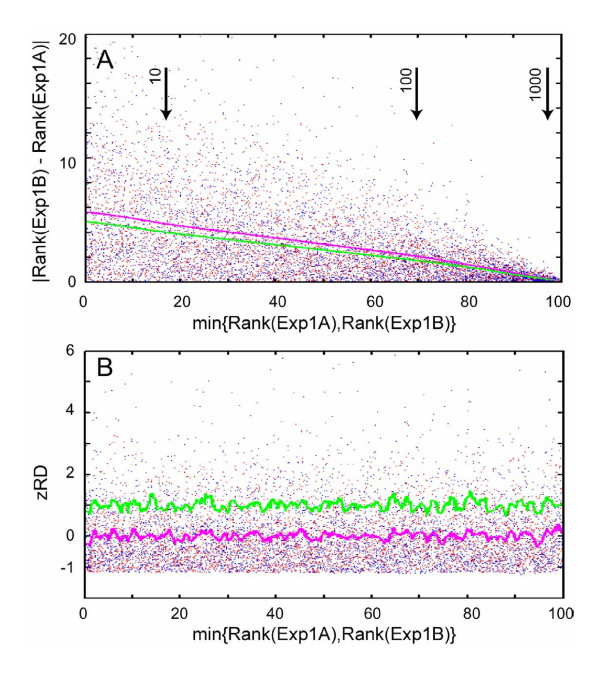
**Effect of positive variation normalization. A **– Increasing rank differences in the comparison Wt-t0b vs Wt-t0a are plotted (in red) against the rank of Wt-t0a. Similarly, increasing rank differences in the comparison Wt-t0a vs Wt-t0b are plotted (in blue) against the rank of Wt-t0b. In the nomenclature of the TOR experiment, Wt and Mu refer, respectively, to strains SH100 and SH121, t0, t1 and t2 refer to 0 h, 2 h and 6 h time points, and a and b indicate the replicates. A sliding window of 100 points was moved by steps of 30 points. At each position, the mean (upper magenta curve) and std (lower green curve) were plotted and the resulting jagged lines were smoothed by a fitting procedure [25]. The vertical arrows indicate the position of 10, 100 and 1000 signal on the 0–100 rank scale. **B **– Distribution of zRD (mean (magenta curve) and std (green curve) are not smoothed).

We first restricted our study to positive variations. As the distribution of positive variations should be the same in both comparisons – Exp1B vs Exp1A and Exp1A vs Exp1B – we plotted the positive variations against rank for both comparisons on a single graph. This revealed that the most salient property of variation distribution, common to all tested measures of variation, is its dependence on the signal rank. This is exemplified for the RD in figure 1A, which shows the absolute value of RD against the minimum of the ranks (i.e., |R_i_(Exp1A) - R_i_(Exp1B)| vs min{R_i_(Exp1A), R_i_(Exp1B)} for gene i). This mode of presentation can be interpreted in either of two ways: as a plot of the positive variations of both comparisons, or as the plot of the positive and the negative variations of a single comparison, with both of variation being represented by a positive number. Whichever interpretation is prefered, it should be underlined that this presentation allows all the gene variations to be taken in account, and ensures the uniqueness of the resulting variation distribution.

We tried to eliminate dependency of positive variation distribution on the signal rank by standardizing variations according to the general formula:


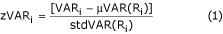


where VAR is to be replaced by any one of the variation units tested (SD, FC or RD). Using this expression, the sample mean and standard deviation (μVAR and stdVAR) were calculated for all genes having a rank within a given neighbourhood of R_i_, the rank of gene i. This notation reflects the fact that the VAR distribution is not gene specific, but rank dependent.

**Figure 2:**
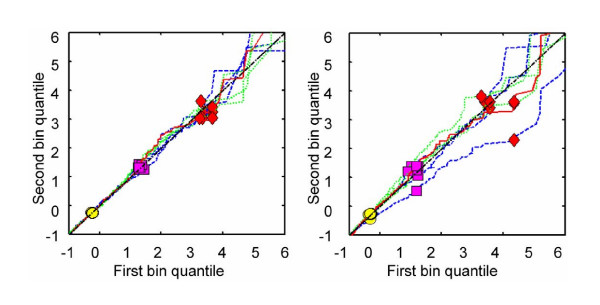
**QQ plots of rRD. A **– Percentiles of zRD distribution in one bin were plotted against corresponding percentiles of another bin. A bin is the set of all genes having a rank in Wt-t0a that falls between two successive decimal values (e.g. between 30 and 40). Blue dashed, green dotted and red continuous lines indicate, respectively, QQ-plots between contiguous bins, between bins three bins apart, and between the first and the last bin. 50th, 90th and 99th percentiles are marked, respectively, by a yellow circle, a magenta square, and a red diamond, **B **– QQ plot of zFC.

Figure 1B shows the results obtained by applying this procedure to the distribution of zRD. In concrete terms, we traced, as shown in figure 1A, two standardization curves, μRD and stdRD, which provide for a given rank the local mean and standard deviation of all the genes with a similar rank. Then, each RD_i _was standardized according to (1). Figure 1B illustrates the beneficial effect of this standardization procedure: first, the mean and the std are no longer dependant upon the rank. Second, the distribution is equalized all along the rank scale, as shown by QQ-plots in figure 2A.

Comparable results are obtained if the standardization is applied to the FC or the SD, but the zRD gives the best results in terms of distribution equalization: figure 2B shows, for example, that QQplots derived from zFC are more erratic than those derived from zRD, which are almost identical to the first diagonal up to the 99th percentile.

The fact that the zRD distribution is independent of rank can be explained by the fact that each gene's zRD_i _follows the same zRD distribution. Therefore, we reasoned that the empirical cumulative frequency distribution, ecfd(zRD), approximates the distribution of zRD for any gene i under the null hypothesis, and we used Fo = 1 - ecfd(zRD), based on a comparison between two replicated experiments, to assign a p-value to any zRD calculated in a comparison between two different biological conditions (the p-value is defined as the probability of zRD of an unchanged gene i to be equal to or greater than the observed zRD_i _under the null-distribution Fo). Because of the very large number of genes present on a chip, the null distribution is sampled a great number of times, generating a quasi continuous set of points that spans a wide range of values. This improves the precision of the Fo curve and allows accurate p-values to be assigned for even large variations.

The entire procedure can then be applied to the comparison Exp2A vs. Exp1A. Because standardization curves constructed on the basis of the two replicates are used in the standardization process, the calculated zRD can be justifiably compared to the null distribution and interpolated on the Fo curve in order to assign a p-value to each gene variation. At this step positive and negative variations can be processed together, although it is necessary to keep track of the actual type of variation, i.e. positive or negative, in order to conduct subsequent analysis.

### Standardization of negative variations

**Figure 3:**
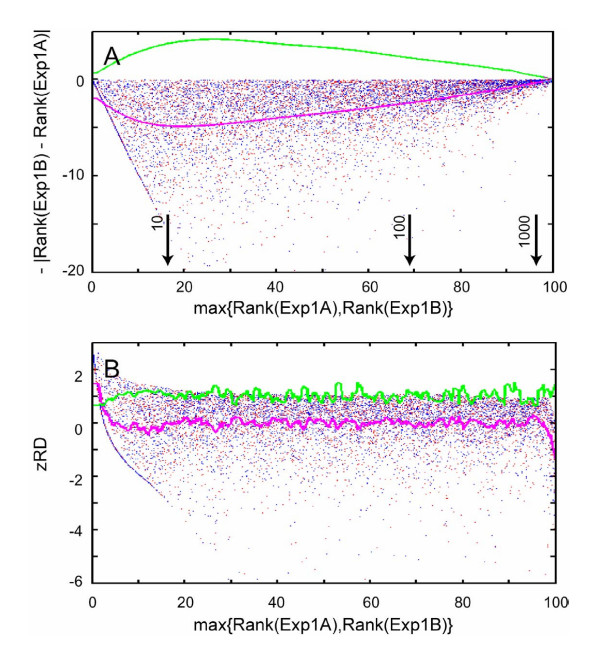
**Effect of negative variation normalization. A **– Decreasing rank differences in the comparison Wt-t0b vs Wt-t0a are plotted (in blue) against the rank of Wt-t0a and decreasing rank differences in the comparison Wt-t0a vs Wt-t0b are plotted (in red) against the rank of Wt-t0b. A sliding window of 100 points was moved by steps of 30 points. At each position, the mean (lower magenta curve) and std (upper green curve) were plotted and the resulting jagged lines were smoothed by a fitting procedure [25]. The vertical arrows indicate the position of 10, 100 and 1000 signal on the 0–100 rank scale. **B **– Distribution of zRD (mean (magenta curve) and std (green curve) are not smoothed).

We also tested to see if it is possible to apply the same standardization techniques to negative variations. In figure 3A, we have plotted the opposite of absolute RD value against the maximum of the ranks (i.e., -|R_i_(Exp1A) - R_i_(Exp1B)| vs max{R_i_(Exp1A), R_i_(Exp1B)} for gene i). It is clear that the weak signals are characterized by a truncation of their variation distribution, as evidenced by the clear alignment of points between ranks 0 and 20. This explains why the standardization procedure applied in figure 3B fails to equalize the variation distribution, and also why the power of the test is lower for down-regulated genes than it is for up-regulated genes (see Discussion).

### False Discovery Rate (FDR), Total Variation (TV) and Sensitivity (S)

**Figure 4:**
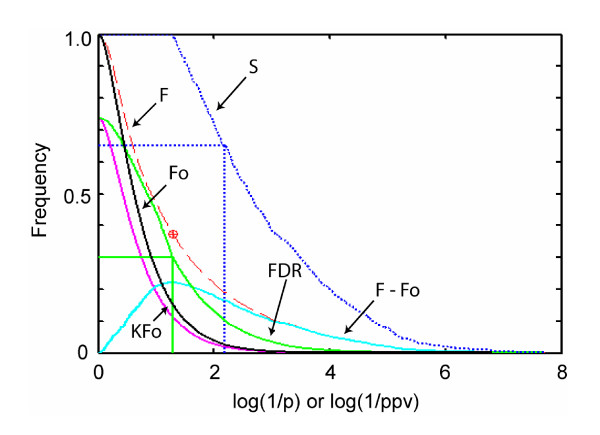
**Selection abacus. **The following functions of x are displayed: F (red dashed curve), KFo (magenta continuous curve), FDR (green continuous curve) and S (blue dotted curve). The crossed circle marks the position at which the maximum of F-Fo (cyan continuous curve) is detected. Interpolation on the FDR curve shows that FDR~30% at S = 100% (green continuous straight lines). Similarly, interpolation on the S curve shows that S~65% at FDR = 10% (blue dotted straight lines). Abscissa x could be either log10(1/p-value(zRD)) in the case of a simple comparison or log10(1/ppv) if replicates are used. All the curves are constrained to be strictly decreasing functions except F-Fo.

Because the test is repeated N times, issues related to multitesting must be considered: the more tests that are performed, the more an outlier outcome becomes probable. In view of this, we first compute the observed distribution of zRD in the comparison Exp2A vs. Exp1A for increased and decreased genes, giving two curves: F_INC _for the positive variations and F_DEC_ for the negative variations (in both cases F = 1 - ecfd (zRD)). Then we plot F_INC _(or F_DEC_) and F_0 _on the same graph, corresponding to the observed positive (or negative) variation distributions and to the expected variation distribution according to the null hypothesis, respectively (figure 4). In the following discussion, the rationale is the same for increased and decreased variations, and F stands for either F_INC _or F_DEC _(same remark for TV, FDR, S, K and N). In most encountered situations, F is on top of F_0_. F(x) gives the probability, for any variant or invariant gene i, of observing a zRD_i _that is at least as high as x, and NF(x) gives the corresponding number of genes. If 0 <= K <= 1 is the fraction of invariant genes, then KNF_0_(x) is the number of invariant genes that have a zRD_i _at least as high as x. As a consequence, NF(x) - KNF_0_(x) is an estimate of the number of variant genes with zRD equal to or greater than x. We can call k the value of x that gives the maximum number of variant genes, such as NF(k) - KNF_0_(k) = max(NF(x) - KNF_0_(x)), and use this value as an estimate of the total variation (TV), that is, the number of truly varying genes. As these quantities must verify the equation N = KN + TV, i.e., N = KN + NF(k) - KNF_0_(k), we can deduce that 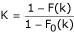.

For each value of x, we estimate the False Discovery Rate,


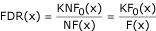


and the sensitivity,


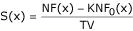


In the context of a transcriptome analysis, p-values reflect how probable it is that a variation reaches or exceeds an observed value. P-values can always be used to rank genes, but the selection of significant variations in the context of multiple testing requires defining significance levels that are far more stringent than 0.01 or 0.05, as used in single testing. FDR and S parameters allow this difficulty to be overcome: for each c used as a potential critical value, S(c) reflects the fraction of truly-varying genes that are selected by zRD>c, and FDR(c) estimates the fraction of selected genes that are likely to be invariant genes. It is therefore possible to plot FDR and S against c, and to construct, for positive and negative variations, what we call a selection abacus (figure 4).

### Analysis of replicates

The simplest example of a replicated experimental scheme is the system {Exp1A, Exp1B, Exp2A, Exp2B}. While it would be tempting to average signals or ranks for each experimental condition and apply the method described above, this is not possible because averaging changes statistics and we have no practical way of obtaining the corresponding empirical null distribution.

In a first round of comparison, we conducted two analyses in parallel by applying RDAM to the first comparison Exp2A vs. Exp1A and to the second comparison Exp2B vs. Exp1B. Based on this first round of comparison, we obtained two p-values for gene i: p1_i _and p2_i_. It could occur that gene i is detected as an increasing variation in the first comparison and as a decreasing variation in the second comparison. In this case, we apply a direction rule to decide on the final direction of variation. We consider simply that the lowest p-value is in favor of its corresponding variation direction, and we set the p-value of the discordant comparison to one. Once we have calculated and possibly corrected the p-values, we construct a new random variable, the product of p-values, ppv_i _= p1_i _× p2_i_. To obtain an unbiased value for ppv, we apply the same procedure to a second round of comparison by exchanging Exp2A and Exp2B between the two comparisons, giving a second ppv value. The direction rule is applied to the two ppv before obtaining the final, averaged ppv.

The advantage of the random variable ppv is the ease of constructing its null distribution. In fact, the cfd(p_i_|H_0_) is a uniform distribution over the interval [0,1]. Therefore, for cases in which two independent comparisons between two sets of duplicates were to be considered, we constructed two sets, U1 and U2, of 100000 points uniformly distributed over the interval [-1,1], to take into account the possibility of increased and decreased variation for each point. These sets were randomized to make them independent in order to model the independence of measurement according to the null hypothesis. This hypothesis states that all variations are due to noise, and that for a particular gene all corresponding p values must be independent. Then, we apply the direction rule to the pair U1, U2 and calculate ppv for genes that are detected as increased. Thus, the F_0 _= 1 - cfd(log10(1/ppv)) curve allows the significance of any value for ppv to be tested. The significance of ppv combines the significance of variation within each individual comparison and the significance of the correlation between these variations. F curve is, as usual, the observed 1 - ecfd(log10(1/ppv)), and we get exactly the same kind of selector abacus, as shown in figure 4. Simulation of the ppv null distribution used exactly the same steps that the analysis process follows, i.e. application of the direction rule and construction of the product of p-values, and resulted in a null distribution model we found appropriate seeing, both with experimental and synthetic data sets, that the observed distribution of ppv matches the null distribution when no variation occurs (data not shown).

The system {Epx1A, Exp1B, Exp2A, Exp2B} allows the construction of two sets of sandardizing curves, one from Exp1 replicates and the other from Exp2 replicates. As these curves are not equivalent, it is necessary to carry out both analyses and then use the more conservative one, whichever has the lower F curve.

The generalization of the entire procedure to more than two replicates is straightforward. For example, with three replicates {Exp1A, Exp1B, Exp1C, Exp2A, Exp2B, Exp2C}, there are 3 × 2 ways of arranging the experiments in order to obtain different sets of comparisons. Each round of comparison gives three p-values for the gene i - p1_i_, p2_i _and p3_i _– and the direction rule is applied the following way: in case the gene i is detected as an increasing variation in the first comparison and as a decreasing variation in the two other comparisons, we compare p1_i _to p2_i _× p3_i _and determine the variation direction. Once we have calculated and possibly corrected the p-values, we obtain the product of p-values for gene i, ppv_i _= p1_i _× p2_i _× p3_i_. As the number of comparison rounds increases very rapidly with the number n of replicates (n!), we simply apply a circular permutation – the circular permutation of ABC consists in the subset of permutations ABC, BCA and CAB – to the replicates inside one of the biological condition which allows the number of rounds of comparison to be restricted to the number of replicates (n).

### Generation of synthetic data

**Figure 5:**
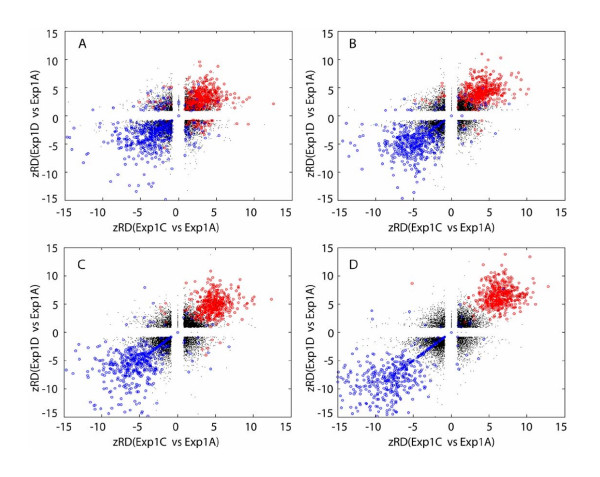
**plot of zRD of the comparison Exp1D vs Exp1A against zRD of the comparison Exp1C vs Exp1A. **Exp1C and Exp1D are synthetic data sets made from the experimental replicates Exp1A and Exp1B (sh100 at t = 0 h). As zRD can be negative, we have plotted positive variations as zRD+2 and negative variations as -zRD-2 to obtain a clear separation of increased and decreased genes. All of the unchanged genes have their zRD set to zero. Red and blue circles mark the genes that received, respectively, an increasing (500 genes) or decreasing (500 genes) additional variation contribution. Black points mark all the genes that did not receive an additional variation contribution. The additional variation p-value was set to 0.30 (**A**), 0.10 (**B**), 0.05 (**C**) and 0.01 (**D**).

In order to test our method, we devised a way of generating synthetic data having similar statistical properties as real biological data. We selected two replicated experiments, Exp1A and Exp1B, as seeds for generating synthetic data, traced standardization curves, and calculated ecfd(zRD) for (Exp1A, Exp1B). We randomly selected half of the genes and exchanged the signals for Exp1A and Exp1B, giving two new data sets Exp1A' and Exp1B'. Random numbers uniformly distributed over interval [0,1] are generated for each gene. Each random number is interpolated on the inverse of the cfd of zRD to assign a random standardized variation zRD_i _to each gene i. New values R_i _are obtained by adding or subtracting to the rank R'_i _of Exp1A' the rank difference RD_i _calculated by applying to zRD_i _the inverse of the normalization function (1). Rank values are finally back-converted into signal values by interpolation of the rank on the graph of signal vs. rank constructed with one of the original data sets. This procedure allows two data sets to be obtained, Exp1C and Exp1D, which are statistically indistinguishable from the original data. To obtain a synthetic data set in which a pre-determined subset of genes receives a significant variation value we can possibly add a second step. We selected in Exp1C and Exp1D a random subset of genes, for example 500 increasing genes and 500 decreasing genes. To these genes, a second random variation value is applied, but instead of drawing random numbers on the interval [0,1], we limit the selection to the interval [0,p]. If we set the limiting p to 0.10, then the variation applied to the subset will have a p-value <= 0.1. For the genes receiving an additional variation contribution, the mean magnitude of zRD that is calculated between the synthetic and the original data is proportional to the magnitude of the applied p-value, as shown in figure 5.

This entire procedure, composed of two successive steps, results in synthetic data sets of high quality, because the generation of data mimics the observed variation of genes. We compared the synthetic data sets Exp1C and Exp1D to the same natural data set Exp1A, and plotted the corresponding zRD of one comparison against the other, as shown in figure 5. We observed that these zRD were independent for genes that had not received an additional variation contribution, but were correlated for genes that had been changed. In general, however, this correlation was not absolute, except for some decreased genes. This phenomenon is explained by the high noise that characterizes weak signals: for such genes, there is a high probability that negative variation makes them reach the minimal rank value (zero). For high signals, there also exists a limit for the rank variation, but the noise is very small, and the truncation effect is not visible. We also observed that a small proportion of genes receiving an increased (decreased) variation contribution could be detected as increased (decreased) in one comparison, but decreased (increased) in another. All of these properties support the realistic nature of the synthetic data generated by our algorithm.

### RDAM performances

We generated several synthetic data sets from the two experiments (Wt_t0a and Wt_t0b) by letting the number of increased and decreased genes equal 0, 100 or 500, and letting the maximum p-value of extra variation equal 0.3, 0.1, 0.05 or 0.01.

**Figure 6:**
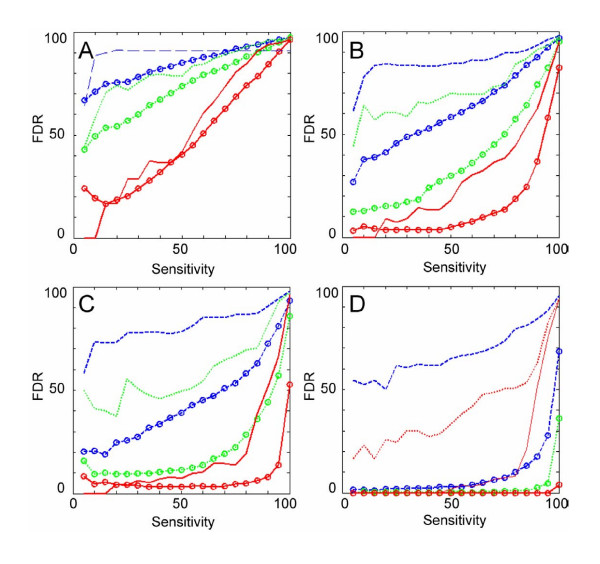
**Performance of the scoring procedure. **Comparisons were made between two groups of 2, 3 or 5 replicates. The first group is composed of synthetic data sets made from the experimental replicates Wt-t0a and Wt-t0b, in which 200 genes (100 increased and 100 decreased) are changed with different strengths (p <= 0.30(**A**), 0.10(**B**), 0.05(**C**) or 0.01(**D**)), and the second group is composed of synthetic data sets without extra variation. Ten comparisons with different combinations of data sets were made, and the resulting mean FDR of increased genes is plotted against given values of S (0.05 to 1, by steps of 0.05). Replicates 2, 3 and 5 are, respectively, plotted with blue interrupted, green dotted, and red continuous lines. Lines marked with circles and without circles refer, respectively, to the results of RDAM and SAM. Only one comparison was used for SAM analysis.

The first question we addressed relates to the effectiveness of our scoring method in discriminating among real variations: does the overall process rank the genes correctly? To address this point, genes were ranked according to their ppv, and the number of hits was computed in a series of sublists of increasing length, selected from the top. From this number of hits, and the number of genes in the sublist, we calculated the real S and FDR. Plotting FDR against S allowed us to visualize the respective effects of the number of replicates, the magnitude of the variations, the number of varying genes, and the direction of variation on the performance of our rank difference method. Figure 6 shows the effect of the first two parameters on the ranking of increasing genes, in this case 100 varying genes. The FDR_50_, defined as the FDR observed when S = 50%, can be used to demonstrate these effects. Increasing the number of replicates improves the scoring performance, but this improvement is strongly modulated by the magnitude of the applied variation. For example, when the number of replicates equals, successively, 2, 3 and 5, the FDR_50 _equals, respectively, 85%, 74% and 41% for small variations (p <= 0.3) and 3%, 0% and 0% for large variations (p <= 0.01). For variations that we consider from our experience to be realistic, i.e., p <= 0.10, the FDR_50 _is equal to 58%, 30% and 5%, respectively. The number of varying genes also has an important impact, since under the same variation conditions, but with 500 increased genes instead of 100, we measured FDR_50 _values of 24%, 8% and 1%, respectively, which represents a mean decrease in the FDR_50 _of 20 percentage points.

**Figure 7:**
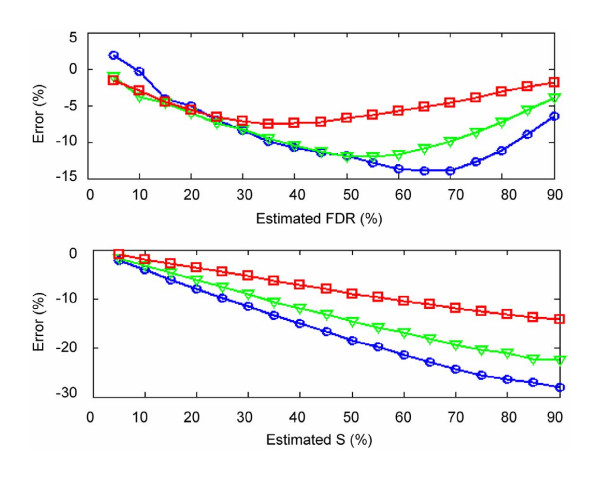
**Error of FDR and S estimators. **Comparisons were made between two groups of 2, 3 or 5 replicates. The first group is composed of synthetic data sets made from the experimental replicates Wt-t0a and Wt-t0b, in which 1000 genes (500 increased and 500 decreased) are changed (p <= 0.10), and the second group is composed of synthetic data sets without extra variation. Ten comparisons with different combinations of data sets were made, and the resulting mean errors of FDR (upper panel) and S (lower panel) for several values of the estimator (5 to 90%, by steps of 5%) are plotted (error is computed by substracting the estimated value from the real value and expressed in relative percentage point difference). Replicates 2, 3 and 5 are marked, respectively, by blue circles, green triangles and red squares.

The second question we addressed is the quality of the FDR and S estimators. The genes were ranked according to their estimated FDR (or S), and the number of hits computed in a series of sublists of increasing length, selected from the top. From this number of hits, and from the number of genes in the sublist, we calculated the mean real FDR and S (figure 7). Both FDR and S are overestimated in this case, except for the point at 5% FDR in the groups with two replicates. If we consider individual comparisons, the distribution of errors has a higher variance for small estimator values. Despite this dispersion of errors in the low FDR range, the absolute number of genes attributed to a faulty category is always negligible and mainly conservative (overestimation of false positives), as shown in Table 1. Figure 7 shows that the ratio between real and estimated S is rather constant, and we found that this ratio was close to the ratio between the estimated and real TV.

Finally, we tested to see whether the independent analysis of positive and negative variations subsequent to standardization was dispensable, or if a one-step procedure could be used instead. In order to illustrate this point, we have constructed, from the experimental replicates Exp1A and Exp1B (sh100 at t = 0 h), a first group of two synthetic replicates having 500 increased and no decreased genes and a second group of two synthetic replicates without changed genes in order to reveal any clear differences that may exist between the one-step and the two-step procedures. Table 2 shows the number of genes selected at several FDR levels when the two competing methods were applied to the comparison between the two groups of synthetic data We can see that with the one-step analysis the number of true positives is lower and the estimate of FDR is largely biased toward higher values relative to the two-step analysis.

### Comparison with SAM on synthetic data

The generation of synthetic data is also a powerful tool for comparing different methods of analysis. As an example, we conducted a systematic comparison between RDAM and SAM. We selected this method because it is popular, easy to use (there exists an Excel add-in), and can be considered as representative of numerous other nonparametric methods, which apply Monte Carlo procedures to estimate the distribution of the statistics used to quantify the relative difference of gene expression. Figure 6 shows that for two and three replicates our scoring procedure generates less FDR than SAM does across the entire sensitivity scale. For five replicates, the scoring procedure of SAM is better only in the low sensitivity (<20%) range. In terms of practical gain, and particularly when experimental costs are considered, the improvement obtained with RDAM is important because we have the same overall ranking quality as SAM but with one replicate less. We also compared the errors made on the estimation of FDR by the two methods, when the nominal FDR equals 20% (Table 3). We concluded that in this particular case FDR estimation was as good in RDAM (mean error of -4 percentage points and extreme error values of -7 and -1 percentage points) than in SAM (mean error of 0 percentage point and extreme error values of -6 and +5 percentage points). Other comparisons show that this conclusion holds true for all other conditions used to generate synthetic data sets (data not shown). However, we observed that SAM estimator was unable to reach the nominal level of FDR detection in case of few replicates and/or small extra variations (for example in case of two replicates and extra variation with p >= 0.10, the smallest FDR estimation delivered by SAM is greater than 20%). In conclusion the large differences in the number of true and false positives found between the RDAM and SAM methods (Table 3) are mainly explained by difference of scoring procedure efficiency between the two methods.

### Analysis of the TOR experiment

When strains SH121 and SH100 were shifted to 37°C, RDAM detected roughly 2300–2500 genes as being either increased or decreased in each strain (column TV, Table 4). Most of these gene variations were caused by the temperature shift and were common to both strains, as shown by the differential analysis which detected only up-regulation of genes as a consequence of TOR2 temperature inactivation: 106 genes at 2 h and 92 genes at 6 h (column TV, Table 4). After 2 hours at 37°C, 19 annotated genes showed significant induction with a 10% FDR, whereas after 6 hours 39 genes were induced (supporting Tables 5,6 and 7 [see additional file 1, 2 and 3]). However, these two groups of genes do not overlap, i.e. the shift to the nonpermissive temperature leads to a subsequent and transient increase in transcription of a small set of defined genes. We note that among these 39 genes, 2 are known to be regulated by the amino-acid-responsive transcriptional activator Gcn4 ([13], see Table 7 in additional file 3). With a selection criterion of 20% FDR, five other Gcn4 regulated genes are detected (CPA2, THI11, SNO1, SNZ1 and PRB1). Therefore, it seems that inhibition of the TOR2-unique function leads to an significant increase in the transcription of known Gcn4 target genes. It is still unclear, however, how the TOR2-unique pathway is connected to nutrient sensing or, vice versa, how nutrient sensing interferes with actin polarization.

### Comparison with SAM on the TOR experiment

We ran our method in parallel with SAM in two situations displaying contrasting transcriptional responses. We tested a first comparison, Wt-t1 vs Wt-t0, which is characterized by a high number of varying genes and a good reproducibility between replicates, facilitating the detection of changes as reflected by the results of RDAM analysis which selected 620 increased genes at S = 50% and FDR = 6%. As SAM does not detect any increased genes at this selection level, we compared results obtained by the two methods at FDR = 10% and observed that among the 817 and 804 genes selected repectively by RDAM and SAM, only 426 were found in common. These results match what we found with synthetic data sets in case of high strength of variation (e.g. p <= 0.01 in figure 6D). We then considered comparisons of biological interest, i.e Mu-t1 vs. Wt-t1 and Mu-t2 vs. Wt-t2, and in this situation RDAM did not select any decreased genes and found only a few increased genes (see Discussion and Table 4). On the contrary SAM failed to detect any genes, either increased or increased.

## Discussion

### Analysis of the TOR experiment

RDAM is a method for identifying genes with changing expression levels using the user-determined FDR and/or S selection parameters. This method was used to study the effects of a thermosensitive mutation of TOR2 in yeast. RDAM succeeded in identifying the few genes that are differentially regulated by the TOR mutation from among the entire mass of genes perturbed by the temperature shift. Recently it has been shown that TOR controls the translation of Gcn4 via the eIF4alpha kinase Gcn2 [14]. Under conditions of TOR inactivation by rapamycin, Gcn4 translation is enhanced, leading to the activation of Gcn4-mediated transcription. Our data also demonstrate that TOR2 inactivation leads to enhanced transcription of Gcn4-controlled target genes (biological results based on RDAM analysis are discussed in a forthcoming paper). Further experiments may show how the TOR2-unique function is integrated into nutrient- (or amino acid-) responsive signaling pathways.

### Normalizing of signal

Apart from randomly-distributed noise, microarrays are also prone to systematic effects that can bias the measurement of signal. All analysis methods are sensitive to systematic bias and include a preliminary normalizing step to make chips comparable. This is a limitation of this kind of approach, because the final result depends on the normalizing procedure used. Considering that all normalizing procedures rely on monotonous transformations that do not change the rank of raw data, we reasoned that if we used a statistics based on rank there would be no need to optimize the normalizing procedure. The rank unit we describe is similar to quantile normalization [15], but does not depend on the signal values of a particular chip as a reference: it can therefore be considered as an invariant. For example, if we focus specifically on the Affymetrix platform, we observe that the signal distribution changes with the different versions of the software: in MAS5, the 50th percentile is around 100, as compared to 1000 in MAS4. In our system, the rank of the genes at the same position in the signal distribution would not change, and would always be roughly equal to 50. This rank unit allows the drawing of plots in which all data are evenly distributed alongdimensions representing a signal. In addition, the linear density of points on the corresponding axes is constant, and the skewness of signal distribution has no effect on the graphical representation.

In our system, all values different from 0 or 100 are assigned to one and only one gene, because ordering of signals always delivers a series of contiguous rank values, even in cases of equivalent signal values. 0 is assigned to all unexpressed genes, as long as a robust method is available to detect them, and 100 to all genes for which the signal is saturated. In Affymetrix technology, especially with the scanner setup presently used, saturation is not a matter of concern and in our analysis, the value 100 is simply assigned to the highest signal. It is a complex problem to identify genes that are not expressed in a given experiment, and we decided to consider as absent only genes having a signal less than zero, as they occur in results delivered by MAS4.

Rank normalization results in transformation of the original signal distribution which is heavily skewed towards low values into a uniform distribution. As a consequence high rank variation could be assigned to small signal variations of weakly expressed genes, and it could be argued that our rank normalization method may bias variation detection towards genes with low signal. By using comparison between synthetic data sets we found no evidence of such a bias (data not shown).

### Systematic usage of duplicates and standardization of variation

Our method has been developed within the framework of hypothesis testing and requires knowledge of the variation distribution for each gene when the null hypothesis is verified. The rationale of our approach considers replicated experiments as precisely representing a system in which all genes follow this hypothesis. However, it has long been recognized that the variation distribution expressed as a ratio or fold change is dependent upon the level of gene expression [16], and we show here that this property subsists when difference of signals or difference of ranks is used to measure variation. In theory it could be possible to use numerous replicates to obtain the empirical variation distribution of each gene. This is not possible for practical reasons, however, and we found that the classical centered-reduced standardization procedure can render variation distribution totally independent of gene expression level, as demonstrated by the QQplot analysis of figure 2A, and allow us to use duplicates to obtain the null variation distribution.

### Independent analysis of positive and negative variations

In the algorithmic implementation of our method, we chose to proceed in two steps and deal with increased and decreased variations independently. First, this ensures that symmetrical comparisons (e.g. Exp1 vs. Exp2 and Exp2 vs. Exp1) give perfectly symmetrical results. Second, even if it were possible to devise another method that would allow one to proceed in one step, it seems more logical to consider increased and decreased variation separately. To clarify this point, a clear distinction must be made between up- or down-regulated mRNAs and increased and decreased variation. Regardless of the experimental points that are being compared, one always observes increased and decreased variations, but these variations have no absolute meaning because one only has to reverse the comparison to change the direction of variation. On the contrary, we can speak of up- or down- regulated mRNAs only when a causal effect exists, such as in a differentiation process or a kinetics or drug assay. In other words, a positive variation observed, for example, between two successive time points in a kinetic can be considered as an up-regulation whatever its mechanism – gene or post-transcriptional regulation – but it is meaningless to invoke any particular form of regulation when comparing, for example, two unrelated cancer tissues. In the case of down-regulated genes, the variation distribution of all weakly-expressed genes is truncated, due to the impossibility of a decreasing signal crossing the zero line. In the case of up-regulated genes, we do not observe the same effect for increasing variation of highly-expressed genes, first because the signal distribution is heavily skewed towards low values, and second because the variance of highly expressed genes is very small (figure 1A). The observation that the reproducibility of variation was lower for down-regulated genes than it was for up-regulated genes [17] is partly explained by this reason, and we were able to demonstrate the statistical difference between up- and down-regulated genes by using synthetic data and observing that all FDR vs. S curves (figure 6) constructed with down-regulated genes were lower than the corresponding curves for up-regulated genes (data not shown). Moreover, we conducted a test showing that the joint analysis of increased and decreased variations degrades the quality of FDR estimation and reduces the number of true positives detected.

### Replicates

We did not try to reduce the amount of raw data when using replicates, and devised a two-step method. A first statistics, the standardized rank difference zRD is constructed on each independent comparison, and p-values are assigned by considering an empirical distribution that matches the null hypothesis. Then a second statistics, the product of p-values ppv, is calculated and p-values are assigned from the null distribution obtained by simulation. Simulation of the null distribution used exactly the same steps that the analysis process follows, i.e. application of the direction rule and construction of the product of p-values, and resulted in a null distribution model we found appropriate seeing, both with experimental and synthetic data sets, that the observed distribution of ppv matches the null distribution when no variation occurs (data not shown). It turned out that this scheme is very flexible and of general applicability: because the second step is rooted in a rigorous statistical method that uses only p-values as input data, it is possible to adapt or to improve the entire process simply by focusing on the first step of p-value estimation. For example, to apply our method to cDNA glass arrays, the only step to be modified would be the variation standardization. Alternatively we could use the segmental approach proposed by Yang and colleagues [18], which is claimed to equalize log ratio distribution, or the variance stabilization method of Huber et al [19], which is efficient in equalizing variation distribution of transformed intensity measurements in both cDNA and oligonucleotide platforms.

### FDR, Total Variation and Sensitivity Estimation

The way in which we estimated FDR is exactly the same as that suggested by B. Efron et al. in their demonstration of the equivalence of empirical Bayes and frequentist approaches (Efron B, Storey J. D. and Tibshirani R. , see equation 3.8 and [20]). We did, however, use another heuristic approach to estimate TV because we observed that the estimator proposed by Storey et al [20] could be very difficult or impossible to calculate when the expression of a small fraction of genes changes. We demonstrated using synthetic data that our estimator was not prone to this type of instability (not shown), and that under realistic conditions (additional variation of p <= 0.10) our estimate was 60%, 65% and 80% of the true TV in the case of two, three and five replicates, respectively. The accuracy of this estimator is obviously dependent on the power of the test, which is itself under the control of the number of replicates. We have also shown that estimated sensitivity was biased by a constant factor that was mostly determined by the error made in TV estimation. Finally, it must be emphasized that the error made in TV estimation has little effect on FDR estimation, as demonstrated by forcing RDAM to use the true TV and K values during the process of synthetic data analysis (data not shown).

### Synthetic data sets

Using the empirical noise distribution observed between two replicates, we devised a method for constructing synthetic data sets. Most published methods add noise to a signal that is supposed to represent the true signal of the gene. We showed here that raw signals without denoising could be used and gave excellent result as judged both by the final distribution of signals and by indirect controls such as the preservation of variation distribution and the possibility of successfully analyzing synthetic data substituted for the original data.

Synthetic data sets are well adapted for judging the respective performances of different analysis methods. To characterize the scoring procedure of a particular method, we used a new type of diagram that plots FDR vs. S, two quantities that relate to the subset of selected genes and that seem better adapted than Receiver Operator Characteristic (ROC, [21]), which relates to both selected and rejected genes (FDR vs. specificity). We proposed using FDR_50_, the FDR at S = 50%, as a comparative index between different methods and showed that RDAM has an FDR_50 _that is 30 percentage points smaller than SAM in the case of three replicates and applied changes with p <= 0.10 (figure 6).

## Conclusions

RDAM is a new statistical method whose performances have been precisely evaluated through extensive analysis of synthetic data sets. When applied to TOR experiment, our method succeeded in finding the few genes of biological interest which were concealed in the mass of varying genes induced by the temperature shift. Comparison with SAM showed that our method obtained the same (if not better) results but with a smaller consumption of chips We conclude that the good quality of the results obtained by RDAM is mostly due to the use of replicates to calibrate the noise and to the quasi-perfect equalization of variation distribution, which is related to the standardization procedure used and to the measurement of variation by rank difference.

## Methods

### Preparation of RNA

Saccharomyces cerevisiae strains SH100 and SH121 [12] were grown overnight in yeast extract peptone glucose (YPD), diluted to an optical density measured at 600 nm of 0.05 (OD600 = 0.05), and grown for an additional 4 hours the next day. The main cultures were then inoculated in YPD medium and grown at 25°C or shifted to 37°C for 2 or 6 hours. All cultures were grown as independent duplicates and were harvested at a final OD600 of 0.8 to 0.9 to minimize the influence of differences in growth phase.

Upon harvesting by centrifugation (2 min, 3000 × g) at 4°C, cells were washed once in ice-cold water, centrifuged again, and the cell pellet was flash frozen in liquid nitrogen. Total RNA was extracted using a hot phenol method essentially as described by Schmitt, M.E. et al. [22].

### Microarray hybridization

Affymetrix™ S98 Yeast Genome GeneChips, containing 6,400 S. cerevisiae (S288C strain) genes and 600 additional probe sets representing putative open reading frames [23], were used throughout this study. Synthesis of cDNA and in vitro transcription of biotin-labeled cRNA, as well as microarray hybridisation, washing and staining procedures, were carried out according to standard protocols as recommended by the manufacturer. Two independent preparations were used for each experimental point.

### Data processing

The scanned microarray images were analysed using the algorithm implemented in MAS 5.0 (Affymetrix, Santa Clara, CA) and the generated raw data were further processed by scripts written in Matlab language (MathWorks, Natick, MA.). SAM analysis [3] of synthetic data was made using version 1.21 of the program [24] with the following default parameters: unlogged data, number of permutations set to 100 and "K-Nearest Neighbors Imputer" used.

Raw data files were uploaded to NCBI's GEO repository under the series number GSE1814 .

## Abbreviations

RD, rank difference; zRD, standardized rank difference; FDR, false discovery rate, S, sensitivity; TV; total variation

## Authors' contributions

DM and MH initiated and conducted the TOR experiment. DM and PD processed probe preparation and chip hybridization. MB developped and tested RDAM method. All authors read and approved the final manuscript.

## Supplementary Material

Additional File 1 Table 5 - Genes found decreased in the comparison sh121 vs sh 100 at 0 h and selected at FDR = 10% Click here for file

Additional File 2 Table 6 - Genes found increased in the comparison sh121 vs sh 100 at 2 h and selected at FDR = 10% Click here for file

Additional File 3 Table 7 - Genes found increased in the comparison sh121 vs sh 100 at 6 h and selected at FDR = 10% Click here for file

## Figures and Tables

**Table 1:** Error on FDR estimation in the case of two replicates and 500 increased genes (p <= 0.1)

**FDR**	**#1**	**#2**	**#3**	**#4**	**#5**	**#6**	**#7**	**#8**	**#9**	**#10**
**5%**	1 (12)	2 (8)	0 (3)	0 (3)	0	0 (0)	0 (0)	0 (0)	0 (0)	0 (2)
**10%**	2 (73)	3 (25)	0 (14)	-2 (37)	-1 (2)	2 (20)	0 (0)	1 (12)	-1 (20)	-2 (20)
**15%**	1 (160)	0 (84)	-3 (68)	-4 (59)	-3 (5)	2 (58)	-4 (41)	-3 (40)	-4 (63)	-4 (57)
**20%**	2 (217)	-6 (157)	-11 (130)	-9 (129)	-5 (65)	0 (134)	-8 (152)	-6 (81)	-11 (146)	-10 (136)

Comparisons were made between two groups of two replicates. The first group is composed of synthetic data sets made from the experimental replicates Wt-t0a and Wt-t0b, in which 1000 genes (500 increased and 500 decreased) are changed (p <= 0.10), and the second group is composed of synthetic data sets without extra variation. Ten comparisons with different combinations of data sets were made. For each set of selected genes we computed the difference between the real number of false positives and the estimated number of false positives. In the first column the FDR estimation is indicated; other columns give the number of increased genes either faultly assigned as true positive (positive difference) or as false positive (negative difference) in each of the ten comparisons (numbered from #1 to #10). In parentheses is indicated the number of genes selected for the given value of the FDR estimator.

**Table 2:** Comparison of one step and two step procedures in the case of two replicates and 500 increased genes

	**One-step analysis**			**Two-step analysis**		
**# Selected**	**# Target**	**Est FDR**	**True FDR**	**# Target**	**Est FDR**	**True FDR**

**100**	75	49	25	88	17	12
**200**	133	58	34	167	23	17
**300**	183	67	39	238	29	21
**400**	229	75	43	298	35	26
**500**	272	80	47	336	44	33

Two groups of data were compared under two conditions. The first group is composed of two synthetic data sets made from the experimental replicates Wt-t0a and Wt-t0b, in which the same subset of 500 genes is increased (p <= 0.10), and the second group is composed of two synthetic data sets made from the same experimental replicates, in which no gene was changed. The one-step analysis consists of processing all of the variations together after the standardization step, leading to the generation of only one F curve, whereas with the two-step analysis the observed variation distributions of increased and decreased genes are considered independently, with each having its own F curve (Finc and Fdec). FDR was chosen so as to obtain a predetermined number of selected genes indicated in the first column. The second and fifth columns (# Target) give the number of true positivesselected. The third and sixth column (Est FDR) indicate the level of selection applied and give an estimate of the FDR. The fourth and seventh columns give the true FDR value, corresponding to the ratio of the number of true positives to the number of genes selected.

**Table 3:** Comparison of RDAM and SAM in the case of two, three or five replicates and 500 increased genes

		**FDR50**		**FDR**		**True Positive**		**False Positive**	
**R**	**p var**	**RDAM**	**SAM**	**RDAM**	**SAM**	**RDAM**	**SAM**	**RDAM**	**SAM**

**2R**	**0.3**	55%	63%	33%	-	4	-	2	-
	**0.1**	25%	49%	21%	-	172	-	45	-
	**0.05**	9%	39%	11%	14%	281	23	35	4
	**0.01**	1%	26%	15%	23%	452	175	77	51
**3R**	**0.3**	42%	47%	17%	-	55	-	11	-
	**0.1**	7%	28%	16%	20%	344	156	64	40
	**0.05**	3%	19%	13%	18%	409	246	60	54
	**0.01**	0%	11%	14%	22%	473	381	80	109
**5R**	**0.3**	17%	21%	18%	25%	261	264	58	88
	**0.1**	2%	4%	15%	20%	455	395	81	101
	**0.05**	0%	2%	16%	20%	472	419	87	106
	**0.01**	0%	0%	19%	19%	474	443	111	105

Comparisons were made between two groups of two replicates. The first group is composed of synthetic data sets made from the experimental replicates Wt-t0a and Wt-t0b, in which 1000 genes (500 increased and 500 decreased) are changed, and the second group is composed of synthetic data sets without extra variation. Results are shown for increased genes. The first column (R), indicates the number of replicates used. The second column (p var), gives the p-value of the extra variation introduced to the changed genes. The third column displays the FDR50, and the fourth column (FDR) the real FDR corresponding to the set of genes selected at FDR = 20%. For the same level of selection, columns 5 and 6 give, respectively, the number of true and false positives.

**Table 4:** Results of selection at S = 50% and FDR = 10%

	**Decreased**					**Increased**				
	
		**FDR = 10%**		**S = 50%**			**FDR = 10%**		**S = 50%**	
	
	**TV**	**Nb**	**S**	**Nb**	**FDR**	**TV**	**Nb**	**S**	**Nb**	**FDR**
**MU vs WT t0**	12	4	30%	7	13%	217	0	0%	196	45%
**MU vs WT t1**	0	0	0%	0	0%	106	19	16%	73	27%
**MU vs WT t2**	0	0	0%	0	0%	92	39	38%	51	11%
**t1 vs t0 WT**	791	311	35%	459	14%	1164	817	63%	620	6%
**t2 vs t1 WT**	231	0	0%	244	53%	233	45	17%	189	38%
**t2 vs t0 WT**	1195	759	58%	641	8%	1275	1074	76%	660	3%
**t1 vs t0 MU**	1158	644	50%	642	10%	1093	856	71%	564	3%
**t2 vs t1 MU**	382	19	5%	351	44%	387	180	42%	226	14%
**t2 vs t0 MU**	1289	725	51%	714	10%	1064	954	84%	543	2%

WT and MU refer, respectively, to strains SH100 and SH121, and t0, t1 and t2 refer to 0 h, 2 h and 6 h time points. TV is the total variation, Nb the number of genes selected, S the sensitivity and FDR the false discovery rate as estimated by RDAM. The second header line gives the selection parameter used.
