# Supplementary material for: Rank Difference Analysis of Microarrays (RDAM), a novel approach to statistical analysis of microarray expression profiling data
Source: BMC Bioinformatics. 2004 Oct 11;5:148. doi: 10.1186/1471-2105-5-148 (PMC526220; doi:10.1186/1471-2105-5-148)
Supplement: Additional File 1 — Table 5 - Genes found decreased in the comparison sh121 vs sh 100 at 0 h and selected at FDR = 10% [file 1471-2105-5-148-S1.doc]

| **SGDID** | **Probe name** | **Description** |
| --- | --- | --- |
| S0000523 | 6909_at | YCL018W beta-IPM (isopropylmalate) dehydrogenase |
|  | 3952_at | 2mic_REP1 2 micron plasmid rep1 protein |
|  | 3953_at | 2mic_D_protein 2 micron plasmid D protein |
|  | 3954_at | 2mic_REP2 2 micron plasmid rep2 protein |

Table 5 – Genes found decreased in the comparison sh121 vs sh 100 at 0h and selected at FDR=10%.
