# Supplementary material for: Rank Difference Analysis of Microarrays (RDAM), a novel approach to statistical analysis of microarray expression profiling data
Source: BMC Bioinformatics. 2004 Oct 11;5:148. doi: 10.1186/1471-2105-5-148 (PMC526220; doi:10.1186/1471-2105-5-148)
Supplement: Additional File 2 — Table 6 - Genes found increased in the comparison sh121 vs sh 100 at 2 h and selected at FDR = 10% [file 1471-2105-5-148-S2.doc]

| **SGDID** | **Probe name** | **Description** |
| --- | --- | --- |
|  | 11273_at | non-annotated SAGE orf Found forward in NC_001133 between 73444 and 73614 with 100% identity. See citation Velculescu, V.E., et al. (1997) Characterization of the yeast transcriptome. Cell 8:243-251 |
|  | 7050_at | non-annotated SAGE orf Found reverse in NC_001134 between 680739 and 680900 with 100% identity. See citation Velculescu, V.E., et al. (1997) Characterization of the yeast transcriptome. Cell 8:243-251 |
|  | 3994_at | similarity to Sauroleishmania NADH dehydrogenase (ubiquinone) chain 5 Found forward in NC_001224 between 3940 and 4167 with 99.122807% identity. |
|  | 3997_at | questionable ORF Found reverse in NC_001224 between 13748 and 14122 with 100% identity. |
|  | 3966_i_at | similarity to T.brucei mitochondrion protein SGC6 Found reverse in NC_001224 between 34032 and 34430 with 88.279302% identity. |
| S0006699 | 3804_f_at | TQ(UUG)L tRNA-Gln |
| S0006479 | 3768_i_at | RDN5-1 5S ribosomal RNA |
| S0006481 | 3770_i_at | RDN5-3 5S ribosomal RNA |
| S0006574 | 3508_f_at | TG(GCC)B tRNA-Gly |
| S0006691 | 3478_f_at | TQ(UUG)B tRNA-Gln |
| S0006692 | 3462_f_at | TQ(UUG)C tRNA-Gln |
| S0006693 | 3436_f_at | TQ(UUG)D1 tRNA-Gln |
| S0006694 | 3439_f_at | TQ(UUG)D2 tRNA-Gln |
| S0006695 | 3405_f_at | TQ(UUG)D3 tRNA-Gln |
| S0006578 | 3358_f_at | TG(GCC)E tRNA-Gly |
| S0006696 | 3376_f_at | TQ(UUG)E1 tRNA-Gln |
|  | 2574_at | Saccharomyces cerevisiae chromosome XVI, complete chromosome sequence. Found forward in NC_001148 between 543577 and 544576 with 100% identity. |
|  | 2382_s_at | Saccharomyces cerevisiae chromosome IV, complete chromosome sequence. Found forward in NC_001136 between 634598 and 635597 with 100% identity. |
|  | 2281_s_at | Saccharomyces cerevisiae chromosome V, complete chromosome sequence. Found forward in NC_001137 between 113004 and 114003 with 100% identity. |

Table 6 – Genes found increased in the comparison sh121 vs sh 100 at 2h and selected at FDR=10%.
