# Supplementary material for: Rank Difference Analysis of Microarrays (RDAM), a novel approach to statistical analysis of microarray expression profiling data
Source: BMC Bioinformatics. 2004 Oct 11;5:148. doi: 10.1186/1471-2105-5-148 (PMC526220; doi:10.1186/1471-2105-5-148)
Supplement: Additional File 3 — Table 7 - Genes found increased in the comparison sh121 vs sh 100 at 6 h and selected at FDR = 10% [file 1471-2105-5-148-S3.doc]

| **SGDID** | **Probe name** | **Description** |
| --- | --- | --- |
|  | AFFX-25srRnaa_at | Z73326 SGD:YLR154C Yeast S.cerevisiae 25S ribosomal RNA corresponds to complement of 4212-7605 in Z73326 (regions a-e represent transcript regions 5 prime to 3 prime respectively) |
|  | 10817_at | non-annotated SAGE orf Found forward in NC_001142 between 283262 and 283426 with 100% identity. See citation Velculescu, V.E., et al. (1997) Characterization of the yeast transcriptome. Cell 8:243-251 |
| S0001700 | 10795_at | YKL217W carboxylic acid transporter protein homolog |
| S0001625 | 10731_at | YKL142W mitochondrial ribosomal protein |
| S0003980 | 10373_at | YLL057C similarity to E.coli dioxygenase |
| S0004044 | 10263_at | YLR054C hypothetical protein |
| S0004319 | 9996_at | YLR327C strong similarity to Stf2p |
| S0004406 | 9905_at | YLR414C weak similarity to YLR413w |
| S0004690 | 9571_at | YMR085W putative pseudogene |
| S0004713 | 9549_at | YMR107W hypothetical protein |
| S0005048 | 8973_at | YNL104C alpha-isopropylmalate synthase (2-Isopropylmalate Synthase) |
| S0005376 | 8591_at | YOL016C Calmodulin-dependent protein kinase |
| S0005660 | 8426_at | YOR134W GTPase activating protein (GAP) |
| S0005912 | 8139_at | YOR385W strong similarity to hypothetical protein YMR316w |
|  | 8067_i_at | non-annotated SAGE orf Found reverse in NC_001147 between 974085 and 974252 with 100% identity. See citation Velculescu, V.E., et al. (1997) Characterization of the yeast transcriptome. Cell 8:243-251 |
| S0006077 | 7914_at | YPL156C weak similarity to YDL010w |
|  | 7560_at | non-annotated SAGE orf Found reverse in NC_001148 between 824685 and 824921 with 100% identity. See citation Velculescu, V.E., et al. (1997) Characterization of the yeast transcriptome. Cell 8:243-251 |
| S0000260 | 7076_at | YBR056W identified by SAGE |
|  | 7043_at | non-annotated SAGE orf Found reverse in NC_001134 between 164451 and 164735 with 100% identity. See citation Velculescu, V.E., et al. (1997) Characterization of the yeast transcriptome. Cell 8:243-251 |
|  | 5507_at | non-annotated SAGE orf Found forward in NC_001137 between 67199 and 67363 with 100% identity. See citation Velculescu, V.E., et al. (1997) Characterization of the yeast transcriptome. Cell 8:243-251 |
| S0003393 | 4862_at | YGR161C hypothetical protein |
| S0003445 | 4778_at | YGR213C involved in 7-aminocholesterol resistance |
| S0003475 | 4763_at | YGR243W strong similarity to hypothetical protein YHR162w |
| S0001113 | 4452_at | YHR071W G1\/S cyclin (weak) |
| S0001138 | 4432_at | YHR096C hexose transporter |
| S0001181 | 4388_at | YHR139C sporulation-specific wall maturation protein |
|  | 4001_at | cytochrome-c oxidase subunit I Found forward in NC_001224 between 20508 and 20984 with 99.790356% identity. |
|  | 4005_at | cytochrome-c oxidase subunit I Found forward in NC_001224 between 23612 and 23746 with 100% identity. |
| S0006486 | 3809_s_at | RDN37-1 35S ribosomal RNA |
| S0006484 | 3765_s_at | RDN25-1 25S ribosomal RNA |
|  | 3068_s_at | Saccharomyces cerevisiae chromosome X, complete chromosome sequence. Found forward in NC_001142 between 425301 and 426300 with 100% identity. |
|  | 2827_g_at | Saccharomyces cerevisiae chromosome XIII, complete chromosome sequence. Found forward in NC_001145 between 371593 and 372592 with 100% identity. |
|  | 2642_s_at | Saccharomyces cerevisiae chromosome XV, complete chromosome sequence. Found forward in NC_001147 between 620016 and 621015 with 100% identity. |
|  | 2644_s_at | Saccharomyces cerevisiae chromosome XV, complete chromosome sequence. Found forward in NC_001147 between 622016 and 623015 with 100% identity. |
|  | 2331_s_at | Saccharomyces cerevisiae chromosome IV, complete chromosome sequence. Found forward in NC_001136 between 1307061 and 1308060 with 100% identity. |
|  | 2265_s_at | Saccharomyces cerevisiae chromosome VII, complete chromosome sequence. Found forward in NC_001139 between 133050 and 134049 with 100% identity. |
|  | 2205_at | Saccharomyces cerevisiae chromosome VII, complete chromosome sequence. Found forward in NC_001139 between 547463 and 548462 with 100% identity. |
|  | 2206_g_at | Saccharomyces cerevisiae chromosome VII, complete chromosome sequence. Found forward in NC_001139 between 547463 and 548462 with 100% identity. |
|  | 2207_s_at | Saccharomyces cerevisiae chromosome VII, complete chromosome sequence. Found forward in NC_001139 between 548463 and 549462 with 100% identity. |

Table 7 – Genes found increased in the comparison sh121 vs sh 100 at 6h and selected at FDR=10%. Two genes known to be regulated by the amino-acid-responsive transcriptional activator Gcn4 are underlined in yellow (YKL217w and YNL104c).
